# The moderating effect of emotional competence on job satisfaction and organisational commitment of healthcare professionals

**DOI:** 10.1186/s12913-021-07234-1

**Published:** 2021-11-20

**Authors:** Elena Stamouli, Sebastian Gerbeth

**Affiliations:** Faculty of Human Sciences, University of Regensburg, Regensburg, Germany

**Keywords:** Emotional competence, Job satisfaction, Organisational commitment, Healthcare professionals, Health promotion

## Abstract

**Background:**

Healthcare organisations, such as hospitals, are largely seen as task-oriented, width different people expected to work in interdependent teams. The objective of this study was to investigate the relevance of individual factors (job satisfaction) and individual competences (emotional competence) for organisational commitment in a sample of healthcare professionals.

**Methods:**

A cross-sectional survey was conducted among 96 healthcare professionals from March to June 2018 in the catchment area of five clinics in Bavaria, Germany. The present research examined the moderating role of emotional competence on the relationship between job satisfaction and organisational commitment using moderated regression analysis and simple slope analysis.

**Results:**

Multiple regression analysis indicated that emotional competence moderated the relationship between satisfaction with the job and commitment to the job. The results showed that healthcare professionals with high emotional competence are able to deal more effectively with dissatisfaction in the workplace so that organisational commitment remains unaffected.

**Conclusions:**

Based on the findings of this study emotional competence of healthcare professionals is important for increasing job satisfaction and commitment to the job. Especially for healthcare professionals whose job satisfaction is low, a high level of emotional competence enables them to maintain a high level of organisational commitment. The findings of the study are discussed at the theoretical level for researchers and practical level for hospital managers interested in fostering emotional competence and improving healthcare professionals’ job satisfaction and their organisational commitment, which ultimately may lead to effective performance.

**Supplementary Information:**

The online version contains supplementary material available at 10.1186/s12913-021-07234-1.

## Background

Prior research findings showed that competent, satisfied and committed employees performed effectively in several domains [1–3]. Among other competences, there is an increasing interest to understand the role of emotional competence (EC) as another significant competence at the workplace [4, 5]. Previous research related to the importance of EC by health professionals as crucial competence which seems to promote adaptive reactions in emotionally charged situations [6]. Mintz and Stoller’s [7] systematic review documents that health professionals need to be able to regulate their own emotions and those of others. Research studies highlighted the impact of EC on job satisfaction [1, 6, 8–10] or on organisational commitment [1, 6, 10]. The purpose of the current study is to investigate the moderating effect of EC on the relationship between job satisfaction and organisational commitment.

EC is defined as a multidimensional set of individual abilities and skills of dealing with own emotions and emotions of others in emotion-related situations [11]. It implies cognitive processes or skills (e.g. perceiving, expressing), but goes beyond these in terms of empathy and regulation [12]. For several years, there is a controversial debate concerning the EC construct, as to whether it should be presented solely in terms of ability, or whether it should account for both ability and personality characteristics [13, 14]. In the meantime, the term “emotional competence” is used as a strongly related term to emotional intelligence (EI) [15, 16]. In the present study, we preferred to use the term “emotional competence” due to its multidimensional framework based on the theory of Stamouli [17], which synthesizes the different models of EC and EI [16, 18–21], excluding components that overlap with personality traits. The construct refers to a four-component-model (see Table 1) used to measure emotional perception, emotional sensitivity, emotional expressivity and emotional management [11]. Understanding the four-component-model of EC Stamouli [11] points to the importance of a rating criterion that has to be fulfilled to describe someone’s behaviour as emotional competent or not. Based on Saarnis´ [21] suggestion about the relevance of one’s own self-efficacy (behaviour according to own targets and values) as criterion to understand the level of someone’s EC Stamouli [11] adds that a bilateral rating criterion (subjective and situational context) is necessary. The subjective context may clarify the motives of certain behaviour, but the situational context seems to be just as meaningful as a rating criterion to describe a behaviour or action as competent. Understanding EC means to take into account that its impact can vary depending on the organisational and work situations.

**Table 1:** Dimensions of emotional competence by Stamouli (2009)

The Emotional Competence Questionnaire	include following skills …	Number of items
**1. Perception of own emotions**		**11**
Attention to one’s own emotions (AE)	to perceive own emotions.	4
Clarity of emotional perception (CP)	to understand own emotions and the own mood.	7
**2. Perception of the emotions of others**		**4**
Perspective taking (PER)	to take over the perspective of someone else and to consider various points of view.	4
**3. Expressivity of emotions**		**16**
Trust in one’s own expressivity (TE)	to trust the own expressivity and the level of wellbeing is in the center of attention.	4
Positive expressivity (PE)	to feel and express respective emotions of happiness and joy.	6
Negative expressivity (NE)	to express and communicate negatively felt emotions.	6
**4. Emotional management**		**10**
Reflective handling of emotions (RE)	the reflexive reprocessing of emotional incriminating situations.	7
Influencing one’s own emotions (IN)	to stop unpleasant emotions and to emphasize positive aspects.	3

Referring to the job characteristics theory of work attitudes and performance [22], organisational commitment and job satisfaction are determined as important work outcomes. Organisational commitment is defined as “the totality of internalized normative pressures to act in a way that meets organisational interests” ([23], p.418). Based on the three-component model of organisational commitment by Meyer and Allen [24] there is a distinction between affective, continuance and normative commitment. Referring to the purpose of our study we concentrate explicitly on the component of affective commitment that describes an “emotional attachment to, identification with, and involvement in the organisation” ([24], p.67). Previous research to the field of organisational commitment has focused mainly on outcomes of relevance for employers. In contrast, a growing body of research exists examining the links between organisational commitment and relevant outcomes for employees including stress and work-family conflict, job satisfaction and work performance [25–27]. Considering the role of emotions in work situations (e.g. stress, anger) and their effects on employees, we expect that additional individual factors, such as EC, moderate the relationships between organisational commitment and relevant work outcomes for employees.

Job satisfaction has been broadly recognised in both academics and industry. The relationship between job satisfaction as work outcome for employees and organisational commitment has been repeatedly examined in recent research [28–32]. Nevertheless, there are only few studies that examine additional individual factors (e.g. EC) that have an effect on the two concepts [6, 10, 33]. For better understanding and assessing the construct of job satisfaction, Weiss [34] proposed three approaches to examine job satisfaction as a result of: evaluative judgments about jobs, affective experiences at work, and beliefs about jobs. We focus on the approach relating to employees’ job satisfaction as an affective reaction to the job. This type of reaction refers to the extent a person likes her/his job. It may be considered as the “emotional state resulting from the appraisal of one’s job as achieving or facilitating the achievement of one’s job values” ([35], p.1342) and these values are compatible with one’s needs. Bowling et al. [36] posited the importance of job satisfaction as a board construct which is associated with a complex set of interrelationships of tasks, roles, responsibilities, interactions, incentives, and rewards.

### Emotional competence in healthcare

Academic literature indicates that healthcare professionals work in a high-stress environment [37, 38]. Ashkanasy et al. [39] elaborated that EC can influence the reaction to stress in the work environment. Humpel et al. [40] added that the ability to monitor one’s own, and others feelings, and emotions and use this information to guide one’s thinking and behaviour, would seem a useful competency in dealing with work stress. Additionally, EC is fundamental to reduce the risk of burnout among healthcare professionals [41–43]. Thereby, the triggers for work stress are not only organisation-related, such as the pressure to reduce costs, increase quality and reduce risks, but are also found on the intrapersonal as well as interpersonal level. Especially in healthcare, work stress is characterised by the interpersonal involvement with patients and carers [44] and the necessity to earn their trust. Healthcare professionals often have to deal with aggressive, introverted and devoted patients, who sometimes only cooperate insufficiently or who reproach, because their recovery does not proceed as desired [45]. In addition to that, carers and patients often mistrust healthcare professionals. Patients and their carers tend to ask the same or similar questions repeatedly and thus strain the patience of health professionals. According to Giesenbauer and Glaser [46], dealing with emotions and feelings is an essential part of caring and can influence the interaction with patients positively. However, Kim, Kim and Byun [47] argue that the healthcare professionals try to gain an emotional distance as a protective mechanism towards the patients to not get overwhelmed by negative feelings. The gap between social, work demands and protective mechanisms in form of individual skills and competences often leads to difficulties that healthcare professionals have to deal sufficiently within stressful emotional situations. Dealing with one’s own or others’ emotions (for example those of patients) is part of the work of healthcare professionals and demonstrates the relevance of individual EC for coping with these work tasks [4]. Physicians and professionals need EC to meet the requirements of the healthcare profession [48, 49].

Our basic hypothesis is that EC contributes to synthesize work demands (here as organisational commitment) and individual needs (here as job satisfaction).

## Methods

In light of the literature reviewed, the following hypotheses were formulated:

### Hypotheses

1. H1: Emotional competence is positively related to organizational commitment and job satisfaction among clinical healthcare professionals.

2. Dimensions of EC, namely attention to one’s own emotions (H2), clarity of emotional perception (H3), perspective taking (H4), trust in one’s own expressivity (H5), positive and negative expressivity (H6 and H7), reflection of emotions (H8), and influencing one’s own emotions (H9), moderate the relationship between job satisfaction and organisational commitment such that a low level of job satisfaction has less impact on organisational commitment among physicians reporting higher levels of EC.

### Research design

The present study is based on sectional survey research design in which data is collected at one point in time from a sample selected to symbolize a larger population across different clinics for Psychosomatic and Psychiatry Medicine in Bavaria (Germany).

#### Sample and sampling strategy

Data was collected among 96 healthcare professionals from March to June 2018 in the catchment area of five clinics and polyclinics for psychosomatic and psychiatry medicine (Danuvius-Klinik in Pfaffenhofen and Ingolstadt, Klinikum rechts der Isar and the Clinics in Freising and Donaustauf). All clinics and polyclinics are located in Bavaria in Germany. The main cooperating partner of this study works at the University Clinic (Klinikum rechts der Isar: KRI) of the Technical University in Munich and especially, at the clinic and polyclinic of psychiatry and psychotherapy. Therefore, ethic approval was granted by the ethic commission of the University Clinic (KRI) of the Technical University in Munich. In total, 96 healthcare professionals attended the study. In order to gather material about age, gender and years of experience working in a hospital an informed consent form was gathered before data collection.

The majority of the respondents were women (66.7%). 37.5% of the respondents were physicians while 62.5% were nurses. 24% of the respondents were in a leading position in their clinic. A total of 68.8% of healthcare professionals had permanent employment. 55.2% of healthcare professionals were over 40 years old. Thereby, most of the respondents (37.5%) had more than twenty years of work experience while 20.8% had ten to twenty years, 33.3% had less than ten years. Only 4.2% stated that they had less than one year of work experience.

#### Measures

##### Organizational commitment questionnaire (OCQ)

Allen and Meyer [50] define commitment as a psychological state that portrays not only an individual’s association with an organization but also his/her decision to continue the relationship in the organisation. In this research, organisation commitment scale is used as an instrument to measure the workplace attitude of an employee. The scale used is the German version of the OCQ and consists of 15 items [51]. Each item has five elective responses, which are recorded on a five-point Likert scale ranging from strongly disagree to strongly agree. A sample item is “I am willing to put in a great deal of effort beyond that normally expected in order to help this organization be successful”. The internal consistency for this scale was 0.91.

##### Job satisfaction scale

Job satisfaction can be described as the internal involvement that how they feel about the nature of their jobs and tasks at the workplace [52]. Job satisfaction was measured using a 37-item measure developed by Fischer and Lück [53]. Items were presented on a five-point Likert type scale. Sample items from the scale included, ‘I’m quite happy with my job for the time being.’, ‘Are you satisfied with the payment? ’ and ‘I can arrange and plan my work myself.’. In the present study, the internal consistency was 0.95 (for the factors internal consistency ranged from .78 to .89).

##### Multidimensional emotional competence questionnaire (MECQ)

A self-assessed questionnaire was used to measure the level of EC among healthcare professional using eight dimensions of Stamouli [11]. In the development of the measuring instrument, a literature review was conducted to identify the dimensions of EC from previous studies in the field of emotional intelligence and EC. From each study, a list of dimensions was created. Using a Delphi technique, the clinical behaviour and physicians’ expert opinions were used in completing this list. The eight most common EC dimensions were chosen for inclusion in the questionnaire. These included: “attention to one’s own emotions”, “clarity of emotional perception”, “perspective taking”, “trust in one’s own expressivity”, “positive expressivity”, “negative expressivity”, “reflective handling of emotions”, and “influencing one’s own emotions” (this model can be aggregated theoretically in four high-order dimensions: perception of own emotions, perception of the emotions of others, expressivity of emotions and emotional management). This questionnaire had 41 items (Table 1). The answering of the items can be done with the help of a five-point Likert type scale, which is graded into “does not apply, does not apply much, does partly apply, does rather apply, does apply”. The internal consistency was between α = .64 and α = .83 for the eight dimensions of EC.

#### Procedure

For the purpose of gathering data, the researcher visited five clinics of psychiatry and psychotherapy in Bavaria (Germany). All procedures performed in this study were in accordance with the ethical standards of institutional research committee (Ethikkommission des Klinikum rechts der Isar) and with the 1963 Helsinki declaration and its later amendments. The dual permission was obtained firstly from the Ethics Commission and secondly from healthcare professionals individually. Data were obtained from healthcare professionals, who agreed to participate. The questionnaire was designed to take about 20–30 min completion time.

#### Analysis

All data were analysed using SPSS 25 and the PROCESS macro for SPSS [54]. The effects of gender and age were controlled for by including them as covariates in the moderation analysis in the PROCESS macro. The researchers determined the significance level at the rate 0.05, and 0.01. The missing values were checked prior to further statistical analysis. Respondents with missing data were excluded from further analysis. A collinearity analysis was carried out to check the prerequisites for a regression analysis, which showed no signs of collinearity (tolerance factor > .10 and variance inflation factor < 10 [55]). For moderation, the PROCESS plugin was used to test the hypothesis.

## Results

Table 2 shows descriptive statistics and Pearson correlations among study variables. Cronbach α coefficients indicate that there is satisfactory internal consistency of EC and the scales of job characteristics (job satisfaction and organisational commitment). On average, respondents reported experiencing a level of job satisfaction of 3.49, EC presents a range of means from 4.41 to 2.94 and organisational commitment of 3.33. Age was related to negative expressivity (*r* = 0.27, *p* < .01) and reflection of emotions (*r* = 0.20, *p* < .05) while gender was not related to any of the study variables.

**Table 2:** Descriptive Statistics: Means (M), Standard Deviations (SD), Cronbach α and Correlations

			Emotional Competence (EC)								
Subscales of Job Satisfaction (JS)	*M (SD)*	*α*	AE	CP	PER	TE	PE	NE	RE	IN	OC
Self-realization	3.81 (.77)	*.89*	.16	.09	−.15	−.21*	−.18	−.00	.04	−.07	.28**
Resignation	3.51 (.71)	*.78*	.15	.20*	−.10	−.02	−.10	−.10	.20	.06	.21*
Salary	3.13 (.86)	*.82*	.05	.09	−.17	−.10	−.24*	−.08	−.02	.07	.41**
Workplace	3.53 (.77)	*.87*	.05	.04	−.13	−.16	−.17	.01	.05	−.11	.38**
Other reasons	3.48 (.71)	*.77*	.15	.11	−.12	−.05	−.18	−.06	.02	.06	.52**
Job Satisfaction (Total score)	3.49 (.65)	*.95*	.12	.10	−.17	−.12	−.16	−.09	.04	−.02	.40**
*Organisational Commitment (OC)*	3.33 (.72)	*.91*	.01	.02	.04	−.11	−.04	−.01	.00	.23*	
*M (SD)*			4.41 (.63)	3.92 (.60)	3.65 (.59)	2.94 (.93)	3.46 (.60)	3.00 (.68)	3.47 (.67)	3.19 (.69)	
*α*			*.64*	*.73*	*.67*	*.83*	*.66*	*.82*	*.79*	*.64*	

Notes: AE: “attention to one’s own emotions”, CP: “clarity of emotional perception”, PER: “perspective taking”, TE: “trust in one’s own expressivity”, PE: “positive expressivity”, NE: “negative expressivity”, RE: “reflective handling of emotions”, and IN: “influencing one’s own emotions”

(i) *p < .05, **p < .01

(ii) *N* = 96

(iii) *α =* Cronbach α

Table 2 shows that the most subscales of EC were not significantly related to organisational commitment. An exception is the dimensions influencing one’s own emotions (*r* = 0.23, *p* < .05). Therefore, H1 is only partial supported. Clarity of emotional perception (*r* = 0.20, *p* < .05), trust in one’s own expressivity (*r* = − 0.21, *p* < .05) and positive expressivity (*r* = − 0.24, *p* < .05) were correlated with factors of job satisfaction. Job satisfaction was positively correlated with organisational commitment (*r* = 0.40, *p* < .01). Especially satisfaction with salary and the workplace had a positive relationship with organisational commitment (*r* = 0.41 and 0.38, *p* < .01).

The effects of job satisfaction and perceived EC on organisational commitment are represented in Table 3. As shown in model 1, multiple regression analysis, testing the main effects model, yielded a significant and positive regression of job satisfaction on organisational commitment (b = 0.48, *p* < .01).

**Table 3:** Moderation analysis for the effect of EC for the impact of Job Satisfaction on Organisational Commitment

	Organisational Commitment (dependent variable)																	
	Model 1		Model 2		Model3		Model 4		Model 5		Model 6		Model 7		Model 8		Model 9	
Variables	b	SE	b	SE	b	SE	b	SE	b	SE	b	SE	b	SE	b	SE	b	SE
Gender	−.07	.16	−.05	.15	−.05	.16	−.07	.15	−.09	.16	−.05	.15	−.04	.15	−.07	.16	−.09	.15
Age	−.05	.08	−.07	.08	−.08	.09	−.04	.08	−.04	.08	−.05	.08	−.06	.09	−.06	.08	−.08	.08
Job Satisfaction	.48**	.11	.55**	.12	.49**	.12	.61**	.11	.54**	.12	.59**	.11	.48**	.11	.48**	.12	.51**	.11
Attention to one’s own emotions (AE)			−.02	.12														
***Job Satisfaction x AE***			−.42*	.18														
Clarity of emotional perception (CP)					−.00	.13												
***Job Satisfaction x CP***					**−.19**	.19												
Perspective taking (PER)							.23*	.11										
***Job Satisfaction x PER***							−.52**	.14										
Trust in one’s own expressivity (TE)									−.01	.07								
***Job Satisfaction x TE***									−.26*	.12								
Positive expressivity (PE)											.05	.12						
***Job Satisfaction x PE***											−.58**	.16						
Negative expressivity (NE)													.06	.11				
***Job Satisfaction x NE***													−.40*	.16				
Reflective handling of emotions (RE)															.01	.11		
***Job Satisfaction x RE***															**−.13**	.18		
Influencing one’s own emotions (IN)																	.25*	.10
***Job Satisfaction x IN***																	−.38*	.17
F	6.23**		4.96**		3.96**		7.73**		4.56**		7.10**		5.20**		3.70**		5.62**	
R^2^	.18		.23		.20		.32		.22		.37		.25		.19		.26	
ΔR^2^	.17**		.05*		.01		.12**		.05*		.11**		.06*		.01		.05*	

Note: *N* = 87 due to missing data, b = regression coefficient, SE = standard error of the regression coefficient, F = F-test, R^2^ = coefficient of determination, ΔR^2^ = additional R^2^* = *p* < 0.05, ** = *p* < 0.01

**Fig. 1:**
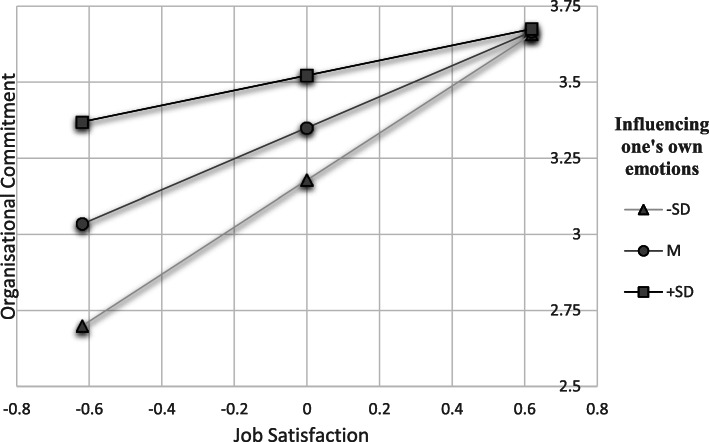
Simple slope analysis – influencing one’s own emotions as moderator of the relationship of job satisfaction on organisational commitment

**Fig. 2:**
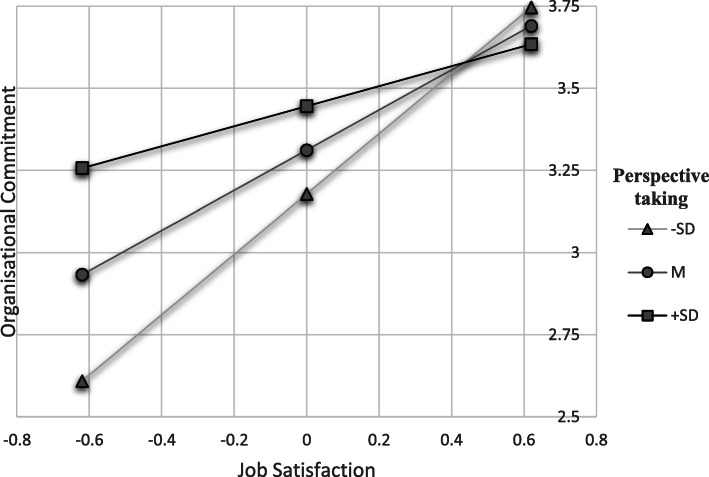
Simple slope analysis – perspectiv

H2 to H9 predicted that subscales of EC would moderate the relationship between job satisfaction and organisational commitment. As shown in model 2–9 (see Table 3), there are significant interaction terms between job satisfaction and attention to one’s own emotions, perspective taking, trust in one’s own expressivity, positive and negative expressivity, and influencing one’s own emotions (b = − 0.26 to −.58, *p* < .05) and explained variance in the model due to interaction effect beyond the main effect (ΔR^2^ = 0.05 to 0.12, *p* < .05). Therefore, H2, H4, H5, H6, H7 and H9 are supported. Simple slope analysis [56] was performed taking into consideration high (one standard deviation above the mean) and low (one standard deviation below the mean) levels of the moderator variable. In supplementary material 1 all simple slopes analyses of the moderation effects are presented. Figures 1 and 2 present the effects concerning influencing one’s own emotions and perspective taking. In terms of influencing one’s own emotions as well as perspective taking, health professionals with high job satisfaction also achieve high organisational commitment regardless of their EC. However, if job satisfaction is low, health professionals with a high level of perspective taking and influencing one’s own emotions manage to show more organisational commitment than their colleagues with low levels. For the other related subscales of EC, these effects for low job satisfaction were similar (Figs. 1 and 2).

Regarding clarity of emotional perception and the reflection of emotions no significant interactions terms were found (see Table 3) and therefore, H3 and H8 are not supported.

## Discussion

The anticipation was that the EC of healthcare professionals would be positively related to organisational commitment. Furthermore, it was expected that EC would be related to job satisfaction. There is some justification for this, as our literature review showed that majority of the researchers confirmed the importance of EC in healthcare organisations. Acquiring knowledge about the effect of each dimension of EC can help organisational leaders to deal effectively with dysfunctional behaviors and make difference in fostering both the strongest.

predictor and the weakest predictor of healthcare professionals’ demonstrating the contribution of each dimension of EC towards job satisfaction and commitment to their healthcare organisations. The results support that one of the dimensions of the construct of EC (influencing one’s own emotions) is significantly positively related to organisational commitment of healthcare professionals. Findings of the study conducted by Tanjour [57] also showed evidence for a positive relationship between EC and job commitment, investigating a sample of 234 academic staffs from selected schools by the Christian-Albrechts-University in Kiel. The findings further revealed that all components of EC are positively associated with affective commitment. Additionally, it was found that the work experience of the academic staff is positively correlated with EC and job commitment.

With reference to the relationship between EC and job satisfaction research results reported that there are significantly positive correlations between three dimensions of EC and factors of job satisfaction. Duchon and Plowman [58] found that the reflection of one’s own emotions by interacting at the workplace suggests work that offers workers a sense of delight and unites workers to the larger good. Thus, reflection, influence and regulation of emotions would positively predict job satisfaction of healthcare professionals. These results confirm the correlation between EC and job satisfaction among 120 nurses in Accra, Ghana [59]. In this vein, job satisfaction appears somewhat consequential to EC [59]. The observation, that there is a significant positive correlation between EC and job satisfaction supports previous evidence in this area [60–64].

Fry, Vitucci and Cedillo [65] and Rego and Cunha [66] reported that workers’ job satisfaction is positively related to workers’ organisational commitment. Based on the regression analysis in our study we also found evidence for the positive relationship between job satisfaction and organisational commitment. The further hypothesis in our study suggests that EC moderates the relationship between job satisfaction and organisational commitment. Multiple hierarchical regression analyses were computed focusing especially the dimensions of EC. Except for clarity for emotional perception and reflective handling of emotions, our results supported the hypotheses that healthcare professionals with high EC are capable of dealing more effectively with the dissatisfaction at work, so that organisational commitment remains undiminished. Especially perspective taking and influencing one’s own emotions were detected as important dimensions of EC moderating the relationship between job satisfaction and organisational commitment. In other words, healthcare professionals who can influence their emotions and are able to change their perspective in emotional situations, show a high level of commitment to their job regardless of whether they are satisfied with their profession or not. Likewise, the findings of the study conducted by Kim and Liu [6] among 137 Chinese newcomers in Hong Kong showed that EC significantly moderated the relationship between taking charge and job performance, such that taking charge was positively related to job performance only when newcomers’ EC was high.

Understanding the factors that influence organisational commitment is crucial to account of both, organisations, and their employees. Previous studies examined the influence of job satisfaction on organisational commitment [67, 68]. None of the studies has researched the moderating effect of the multidimensional framework of EC in this context. The results of the present study suggest, that when healthcare professionals are highly emotional competent, they are highly committed to their organisations even if they are sometimes not satisfied. While jobs are quasi-static (do not change daily but are relatively constant), emotional situations are more dynamic, especially, if individuals with different levels of EC interact with each other. Findings from the present study indicate despite having high perceptions of job satisfaction, individual’s EC likely influences organisational commitment. Results echo with the earlier notion that employees not only needed to feel competent to be motivated to perform [69, 70], but also needed to utilize their competencies at the workplace [71] and EC seems to be the bridge for this. Johnson’s [5] review analysed the importance of EC in the field of healthcare and recommended various ways to implement this important skill into medical educational programs. For example, Teaching-Learning-Arrangements facilitate practical options for healthcare professionals to deal with emotions at work by using their EC and show its importance for this group of professionals [5, 72].

### Study limitations

This study has several limitations that provide opportunities for further research. First, because of the cross-sectional study design, we cannot infer causality. The indirect effect between EC, job satisfaction and organisational commitment cannot be seen as a causal chain. We consider EC to be the more stable variable and consequently assume EC to affect job satisfaction as well as organisational commitment of healthcare professionals rather than the other way around more strongly. Future studies could increase the sample size for the study to have higher external validity. Second, the present study showed that healthcare professionals recognize the significance of more than one dimension of EC dealing constructively with emotional situations at workplace. Nonetheless, it is conceivable that focusing on the multidimensionality of EC may contribute to the holistic development of healthcare professionals’ EC. Longitudinal studies are necessary to analyse the contribution of EC as a multidimensional construct for employees in high emotional labour jobs over time. Third, healthcare professionals reported on their EC, especially on some of its dimensions, a relationship to their job satisfaction as well as to their organisational commitment. Self-reports may be limited by self-enhancement, social desirability bias, and lack of accurate self-knowledge. Future studies with independent measurement are needed to address the problems of a common source and common method bias. Such research could assess healthcare professional’s EC using a performance test, their job satisfaction by field observation for instance during interactions with patients, and healthcare professional’s interpersonal behaviour by patients’ ratings. Fourth, the moderating effect between EC, job satisfaction and organisational commitment may vary across cultural contexts, depending on collectivism, power distance, and other factors. Germany is an individualistic culture. It is not clear whether results would be similar in more high-contact and collectivistic cultures. Further research on the relationship between healthcare professionals´ EC, job satisfaction and organisational commitment in different cultures is needed.

## Conclusions

Moreover, hospital managers must consider the EC of employees when they assign jobs that require organisational commitment in accordance with person-job fit or person–task fit, which is an important human resource practice [73]. Drawing on the evidence established in this study, it can be concluded that healthcare professional’s scores on EC tend to be correlated with their scores on job satisfaction and organisational commitment. This study proves the Nightingale et al. [74] findings that EC has begun to influence healthcare professionals and is a crucial component of medical education [4, 5]. We suggest improving medical education training and programs by helping healthcare professionals to develop emotional abilities in ways that help them to focus on their needs as well as on patients’ needs. Further studies are required to expand the burgeoning evidence base on the relationship between EC and other work-related variables among healthcare professionals. Finally, examining how job satisfaction and EC jointly affect healthcare professional’s outcomes beyond organisational commitment, including patient interaction and trust as well as career success, is also an interesting research direction.

## Supplementary Information

**Additional file 1.**

## Data Availability

The datasets analysed during the current study are available from the corresponding author on reasonable request.

## Abbreviations

EC: emotional competence
EI: emotional intelligence
OCQ: organisational commitment questionnaire
KRI: Klinikum rechts der Isar
