# Supplementary material for: The moderating effect of emotional competence on job satisfaction and organisational commitment of healthcare professionals
Source: BMC Health Serv Res. 2021 Nov 20;21:1257. doi: 10.1186/s12913-021-07234-1 (PMC8605519; doi:10.1186/s12913-021-07234-1)
Supplement: Supplementary file 1 — Additional file 1. [file 12913_2021_7234_MOESM1_ESM.docx]

**Figure 3**. Simple slope analysis –negative expressivity as moderator of the relationship of job satisfaction on organisational commitment

**Figure 4**. Simple slope analysis – positive expressivity as moderator of the relationship of job satisfaction on organisational commitment

**Figure 5**. Simple slope analysis – trust in one’s own expressivity as moderator of the relationship of job satisfaction on organisational commitment

**Figure 6**. Simple slope analysis – attention to own emotions as moderator of the relationship of job satisfaction on organisational commitment
